# Supplementary material for: GNPNAT1 Serves as a Prognostic Biomarker Correlated with Immune Infiltration and Promotes Cancer Cell Metastasis through Stabilization of Snai2 in Lung Adenocarcinoma
Source: Biomedicines. 2024 Jul 4;12(7):1477. doi: 10.3390/biomedicines12071477 (PMC11274686; doi:10.3390/biomedicines12071477)
Supplement: Supplementary file 1 [file biomedicines-12-01477-s001.zip › biomedicines-2976103-supplementary.pdf]

1. Supplementary tables were listed as follow:

Supplementary Table S1. Clinicopathological characteristics for TCGA-LUAD cohort, GSE72094 and GSE13213 dataset

Characteristics	TCGA-LUAD cohort (n=468)	GSE72094 (n=442)	GSE13213 (n=117)
Age (y)			
≤65	227(46.71%)	127(28.73%)	78(66.67%)
＞65	240(49.38%)	294(66.52%)	39(33.33%)
unknown	19(3.91%)	21(4.75%)	-
Gender			
Male	264(54.32%)	240(54.30%)	57(48.72%)
Female	222(45.68%)	202(45.70%)	60(51.28%)
Tumor stage			
T1+T2	423(87.04%)	-	-
T3+T4	60(12.34%)	-	-
unknown	3(0.62%)	-	-
Lymph node stage			
N0+N1	402(82.72%)	-	-
N2+N3	72(14.81%)	-	-
unknown	12(2.47%)	-	-
Distant mentastasis stage			
M0	333(68.52%)	-	-
M1	24(4.94%)	-	-
unknown	129(26.54%)	-	-
Pathological stage			
I+II	374(76.95%)	334(75.57%)	92(78.63%)
III+IV	104(21.40%)	80(18.10%)	25(21.37%)
unknown	8(1.65%)	28(6.33%)	-
Survival status			
Alive	304(62.55%)	298(67.42%)	68(58.12%)
Deceased	182(37.45%)	122(27.60%)	49(41.88%)
Unknown	-	22(4.98%)	-
Follow-up(y)			
< 5	426(87.65%)	393(88.91%)	42(35.90%)
≥5	51(10.49%)	5(1.13%)	75(64.10%)
Unknown	9(1.85%)	44(9.95%)	-

Supplementary Table S2. Clinicopathological characteristics for GSE32863, GSE40491, GSE75037 and GSE115002 dataset.

Characteristics	GSE32863	GSE40791	GSE75037	GSE115002
Histology				
Non-malignant lung	58	100	83	52
Lung adenocarcinoma	58	94	83	52
Age（y）				
≤65	19	-	29	39
＞65	39	-	54	13
Gender				
Male	13	41	24	26
Female	45	53	59	26
Smoking				
Yes	29	80	53	9
No	29	4	30	43
unknown	-	10	-	-
Clinical stage				
I+II	45	92	70	26
III+IV	13	2	13	26

Supplementary Table S3 Sequence of siRNAs used in this study

siRNA	Sequence
si-GNPNAT1#1	5’-CCTTGAATGTCTACCACAA-3’
si-GNPNAT1#2	5’-GCAAGAAACTGAACTGTTA-3’
si-GNPNAT1#3	5’-CGGCAACTCTGATTATAGA-3’
si-Slug#1	5’-UACAUGGAGAUGUCGAGCACCAUTT-3’
si-Slug#2	5’-GCAUUUGCAGACAGGUCAATT-3’

Supplementary Table S4 The sequences of primers for quantitative real time PCR

Gene	Sequence
GNPNAT1	Forward:5’-TGAAACTCCTATGTTTGACCCAAGT-3’
	Reverse: 5’-AGCAGTACAAAGCCTCAAAA-3’
GAPDH	Forward: 5’-CACCCACTCCTCCACCTTTGA-3’
	Reverse: 5’-ACCACCCTGTTGCTGTAGCCA-3’

Supplementary Table S5 The antibodies against protein used in this study

Antibody	Dilution	Company
anti-GNPNAT1	1/1000	Proteintech Group, Inc, China
anti-Vimentin	1/2000	Proteintech Group, Inc, China
anti-E-cadherin	1/20000	Proteintech Group, Inc, China
anti-N-cadherin	1/2000	Proteintech Group, Inc, China
anti-β-actin	1/1000	Proteintech Group, Inc, China
anti-slug	1/1000	Proteintech Group, Inc, China

2. Supplementary Figures were listed as follow:

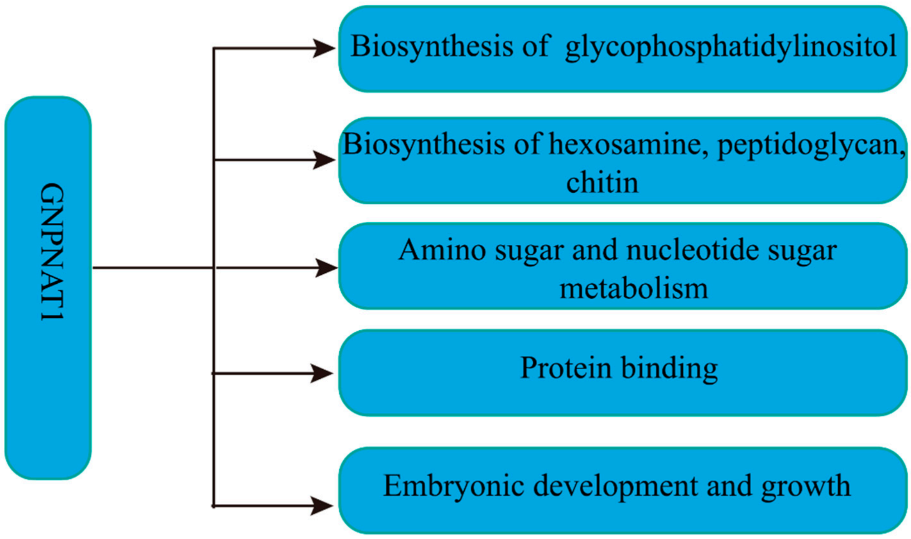

Supplementary Figure S1. The detail function of GNPNAT1.
